# Normalization of Retinal Birefringence Scanning Signals

**DOI:** 10.3390/s25010165

**Published:** 2024-12-31

**Authors:** Boris I. Gramatikov, David L. Guyton

**Affiliations:** Ophthalmic Instrumentation Development Lab, The Wilmer Eye Institute, The Johns Hopkins University School of Medicine, Wilmer 233, 600 N. Wolfe St., Baltimore, MD 21287, USA; dguyton@jhmi.edu

**Keywords:** retinal birefringence scanning, polarization-sensitive systems, ophthalmic screening devices, signal normalization

## Abstract

Signal amplitudes obtained from retinal scanning depend on numerous factors. Working with polarized light to interrogate the retina, large parts of which are birefringent, is even more prone to artifacts. This article demonstrates the necessity of using normalization when working with retinal birefringence scanning signals in polarization-sensitive ophthalmic instruments. After discussing the pros and cons of employing a normalization signal obtained by means of added optoelectronic hardware, the study shifts over and focuses on a numerical normalization method based on merely the *s*- and *p*-polarization components without additional optical or electronic hardware. This minimizes the adverse effects of optical asymmetries, the presence of certain instrumental noise, device-to-device variability, pupil diameter, retinal reflectivity, subject-to-subject variations, the position of the eye in the exit pupil of the device, and even signal degradation by cataracts. Results were experimentally and numerically tested on human data from 15 test subjects and clearly demonstrated the signal standardization achieved by numerical normalization. This is expected to lead to substantial improvement in algorithms and decision-making software, especially in ophthalmic screening instruments for pediatric applications, without added hardware cost. The proposed normalization method is also applicable to other polarization-sensitive optical instruments.

## 1. Introduction

**Figure 1:**
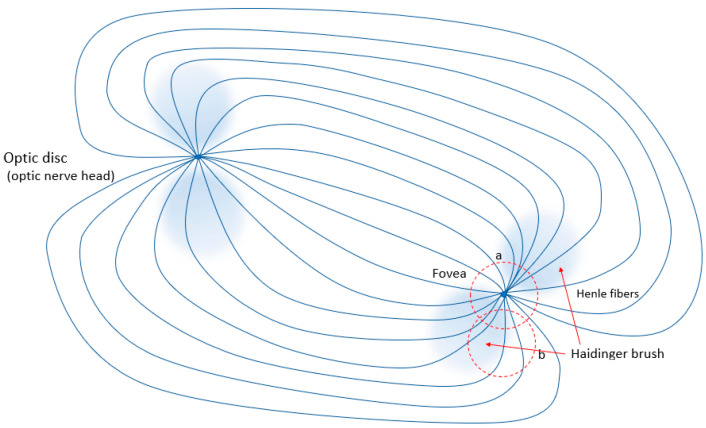
The two birefringent areas on the human fovea. The Henle fibers are the uniquely arranged, radially symmetric nerve fibers surrounding the fovea. They route the visual information to the optic nerve head on the way to the visual cortex. Each circular scan in the vicinity of the fovea is indicated by a red dotted circle. Fast circular scans can establish central fixation (a) or the lack thereof (b).

Retinal birefringence has been studied since the 1980s [1,2,3,4]. Retinal birefringence scanning (RBS) is a method of obtaining two-dimensional information from the retina based on its birefringence. Briefly, a spot of polarized near-infrared light is scanned in a 3° circle to be reflected back from the retinal pigment epithelium, double-passing the retina. The return light (about 1/5000 of the light that entered the eye) is converted to an analog electrical signal and is digitized. In so doing, a full or partial measurement of the 4-component Stokes vector **S** = [S_0_, S_1_, S_2_, S_3_] is obtained. Its first component, S_0_, represents total intensity, whereas S_1_ represents the difference between the vertical polarization component, *s*, and the horizontal component, *p*. Often, it is sufficient to use just S_1_ to measure the change in polarization caused by the retinal birefringence. There are two areas of the retina that exhibit significant birefringence and are of particular interest—the area around the optic nerve head and the area around the fovea (the highest definition part of the retina). Both areas (Figure 1) have been used in specific medical applications. For example, as polarized light passes through the retinal nerve fiber layer surrounding the optic nerve head, the axons in this layer cause a phase shift due to their birefringence. The amount of phase shift is directly proportional to the thickness of the nerve fiber layer. This has successfully been employed in the GDx (short for Glaucoma Diagnosis), a type of scanning laser polarimeter [5,6,7,8]. A more sophisticated enhancement of RBS is the polarization-sensitive form of optical coherence tomography (PS-OCT), providing tissue-specific contrast and adding quantitative diagnostic information [9,10].

RBS around the fovea has become an established method of detecting central fixation (CF) [11,12,13,14]. With it, eye alignment is declared when both eyes (Right Eye, RE, and Left Eye, LE) are fixating at the same time on a small, presented target.

So far, there are two main types of RBS systems. The first type, called here ***1f2f* system**, uses a simple circular scan around the presumed location of the fovea. Its basic principles and some implementations have been reported in detail previously [11,12,15]. Briefly, polarized near-infrared light is reflected from the foveal area in a detectable bow-tie-like pattern of polarization states, allowing localization and eye tracking. The fovea is aimed at the object of regard during fixation. When illuminated with linearly polarized near-infrared light, such as the light emitted by a low-power 785 nm or 830 nm laser diode, the uniquely arranged, radially symmetric nerve fibers (Henle fibers) surrounding the center of the fovea change the polarization state of the light being back-reflected from the underlying retinal pigment epithelium [16,17]. Figure 1 shows the distribution (*Haidinger brush*) of Stokes parameter S_1_ around the center of the fovea. The location of this “brush” can be detected by interrogating the area with a full raster scan [18,19] (which is possible but would be relatively slow), with just a few laser beam spots, or by means of a fast circular scan [11,12,20]. When the goal is to detect merely central fixation, as is the case with pediatric vision screeners [13,21,22,23], circular scanning followed by frequency analysis of the acquired signal is used. When the eye fixates on the center of the scanning circle, the returned scan signal *s*(*t*) has a dominant frequency component *f*_2_, such as twice the scanning frequency *f_s_*, Figure 1, scan **a** (*f_s_*, is the number of revolutions per second that the scanning laser beam makes along the circular trajectory). Alternatively, off-center fixation produces a different signal, of merely the scanning frequency, for example, *f*_1_ = *f_s_*, Figure 1, scan **b**. Off-center directions of gaze generally produce mixtures of the two frequencies and signal traces specific to the direction of gaze, yet the single scanning frequency is always dominant.

**Figure 2:**
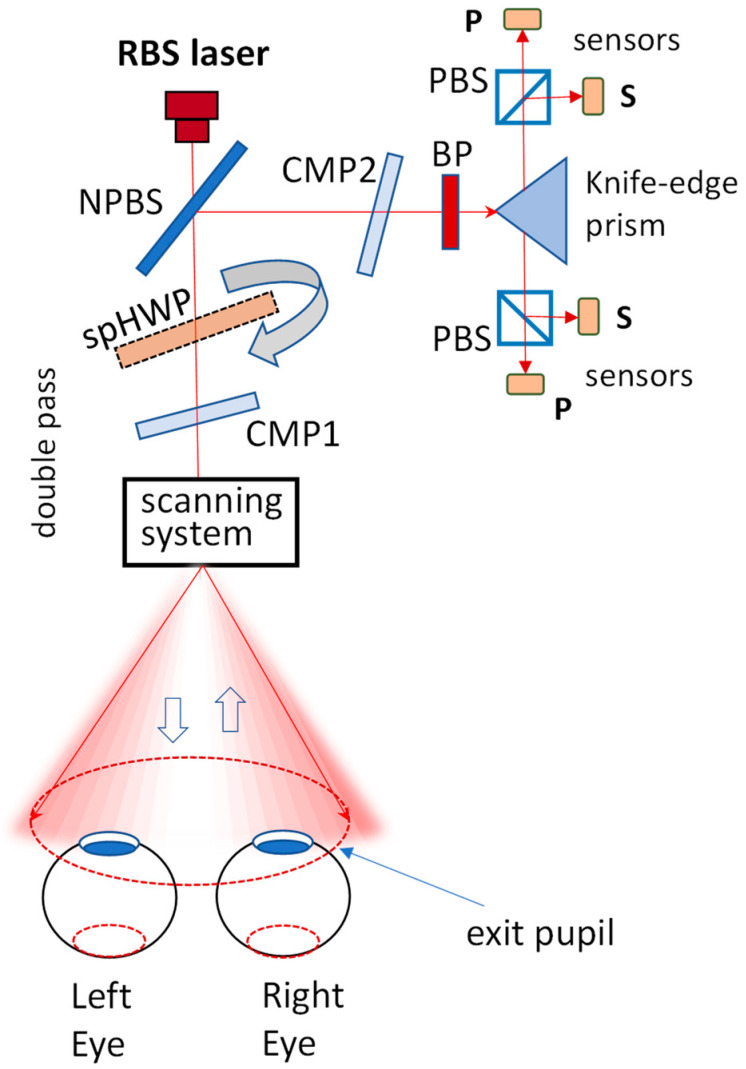
A simplified generic RBS system. The light reflected from the retina is redirected by a non-polarizing beamsplitter (NPBS) to a knife-edge prism, which then separates the two eyes. For each eye, a polarizing beamsplitter PBS splits the light into *s*- and *p*-components, which, after measurement by corresponding sensors, are used to build Stokes parameter S_1_. CMP1 and CMP2 are polarization compensators; BP is a band-pass filter. The spinning half-wave plate spHWP is a polarization rotator, included only in ***spHWP*** systems. Not included are light traps, mirrors, a scanning motor, scanning mirrors, lenses, fixation-attracting optics, etc. Not to scale.

Figure 2 presents a generic RBS system. In it, light reflected from the ocular fundus is redirected to the sensors, and the s- and *p*-components are obtained via a polarizing beamsplitter, one for each eye. Polarization compensators CMP1 and CMP2 are used to compensate for polarization aberrations. The spinning half-wave plate spHWP is present only in spHWP designs, described later, but not in ***1f2f* systems**.

The ***1f2f*** RBS method is fast compared with other scanning methods. It was successfully used for detecting central fixation in early pediatric vision screening devices [13]. Yet, the signal-to-noise level (SNR) here was low and could become comparable with the instrumental noise. This necessitated background subtraction (“flat fielding”), which slows down system performance. As an alternative, in more recent, second-type RBS systems, spatial polarization modulation was introduced [24] incorporating a double-pass half-wave plate (HWP) spinning 9/16ths as fast as the circular scan *f_s_* (Figure 2, including the spHWP). The spinning HWP (***spHWP***) works as a polarization rotator. Upon double-passing through the Henle fibers, the rotating polarization of the incident light modulates the RBS signal and generates half multiples of the scanning frequency upon reflection. The characteristic frequencies now become 2.5*f_s_* and 6.5*f_s_* for central fixation and 3.5*f_s_* and 5.5*f_s_* for para-central fixation. These half-multiple frequency signals double in amplitude and even quadruple in signal strength, as represented by Fast Fourier Transform (FFT) power with 360° phase-shift subtraction, whereas most of the optical background noise (instrumental noise) at whole multiples of the scanning frequency is removed, thus eliminating the need for background subtraction [21,25,26]. This second type of RBS design is termed here a ***spHWP*** system.

In both types of RBS systems, central fixation (CF) is assumed when the spectral power of the scanning signal returned from the retina is above a certain threshold for a characteristic frequency [13] or a combination of frequencies [24]. So far, this has usually been done for each eye separately, and CF has been declared when both eyes pass the same threshold. However, due to optical hardware asymmetries and/or the presence of certain instrumental noise (different for the signals received from the two eyes), device-to-device variability, etc., applying threshold-based decision-making may become imprecise. Furthermore, pupil diameter and retinal reflectivity vary from subject to subject. Our previous studies have also shown that, in one and the same subject, the pupil size changes considerably after the room light is dimmed (which is how most RBS measurements are done). Depending on the accommodation attempt, the pupil becomes at least 50% larger in the time interval between 22 s and 140 s after reducing the ambient light and then contracts again [27]. This can lead to very significant variations in the light reflected from the fundus and, hence, the signal amplitude. Last but not least, the position of the eye in the exit pupil of the device can significantly affect the signal. This we have established during a number of observations and experiments (unpublished).

**Figure 3:**
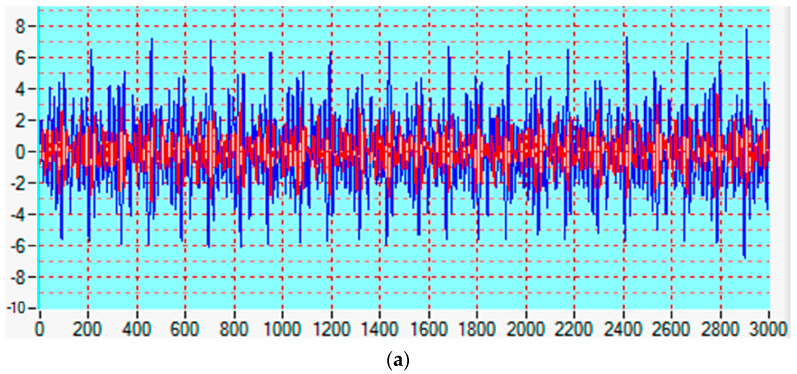

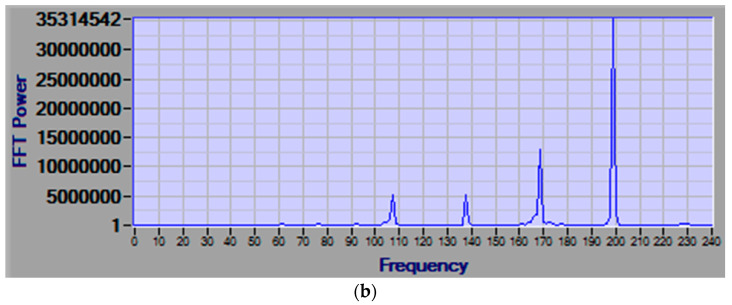
Signals acquired by a typical ***spHWP*** system: (**a**) analog signals (of duration 0.8 s, digitized at 3750 Hz, one eye only): blue for *s*-polarization, red for *p*-polarization; (**b**) FFT power (one eye only, not normalized), in [mV^2^], for Stokes parameter *S*_1_ = *s* − *p*. The frequency is in [Hz].

Figure 3 shows signals acquired by a typical ***spHWP*** RBS system. The upper panel presents the analog signals for one eye as received by the photosensors for *s*-polarization (blue) and for *p*-polarization (red), respectively (for more information on the optical diagram, the reader is referred to Figure 2). In this case, the time epoch being acquired is 0.8 s at a sampling rate of 3750 samples per second. The lower panel of Figure 3 gives the FFT power in [mV^2^] for Stokes parameter *S*_1_ = *s* − *p* (one eye only, not normalized). Since the scanning frequency for a circular scan in this case is 30 Hz (scanning rounds per second), the expected frequency powers would be 75 Hz (2.5*f_s_*) and 195 Hz (6.5*f_s_*) for central fixation (called respectively P25 and P65), and 105 Hz (3.5*f_s_*) and 165 Hz (5.5*f_s_*) for para-central fixation (called, respectively, P35 and P55). As usual, the power spectrum is a mixture of all these frequencies, yet the prevailing frequency power is 195 Hz. Therefore, one can reasonably assume that the test subject is fixating on the target, i.e., central fixation is present. The actual question is, what threshold should be applied to P25 + P65 in order to declare central fixation? It should be emphasized again that signal amplitude depends on many factors and can vary over a wide range.

**Figure 4:**
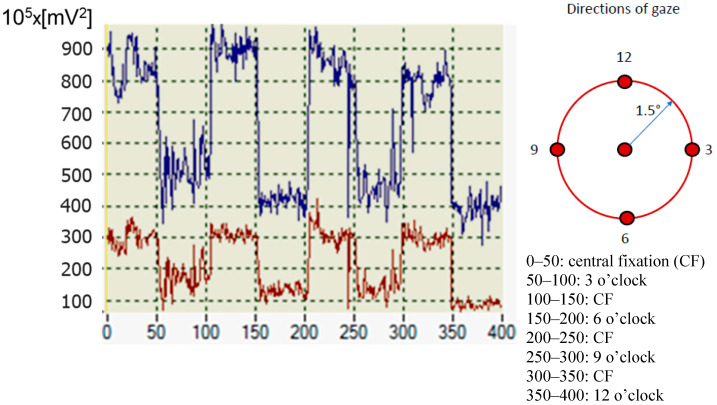
Spectral power P25 + P65 over time, while the test subject was fixating on a target during acquisitions 0–50, 100–150, 200–250, and 300–350. At all other times the subject was fixating on points 1.5° away from the central target (off-CF). The traces are unfiltered and are from a ***spHWP*** system. The x-axis marks the acquisitions, as the data collection progresses. On the power axis (y), one division is equivalent to 10^7^ mV^2^.

All the aforementioned sources of amplitude variations and asymmetry necessitate some kind of normalization. However, choosing the right signal for normalization can be challenging and, in many cases, can negatively impact the reliability of the decision-making logic. Figure 4 illustrates the problem at hand. It shows the traces of the CF-characteristic frequency powers for a ***spHWP*** system: blue for the RE and red for the LE. Each trace represents the combined power P25 + P65 (for 2.5*f_s_* and 6.5*f_s_*, respectively) over a period of ~200 s (400 acquisitions altogether). The spectra were obtained from component S_1_ of the Stokes vector. Each data point is derived from the FFT power obtained from a time-domain RBS signal epoch of duration 200 ms (1000 samples/channel at sampling rate 5 kHz). The test subject (with normal vision) was fixating on a small central target during acquisitions of 0–50, 100–150, 200–250, and 300–350. At all other times, the subject was fixating on points 1.5° away from the central target (off-CF), with the directions of gaze indicated on the right-hand part of the figure. The traces are unfiltered and are from a ***spHWP*** system.

It can be seen that while this test subject’s vision was completely normal and he was responding adequately to instructions to change the direction of gaze, the traces are different, most likely because the two channels have different gain and bias or are in a different position in the exit pupil of the device. Some pupil-size asymmetry, or even cataracts, could also have been the cause. This would prevent the software designer from using simple thresholds applied naïvely to the power spectrum of the scanning signals.

**Figure 5:**
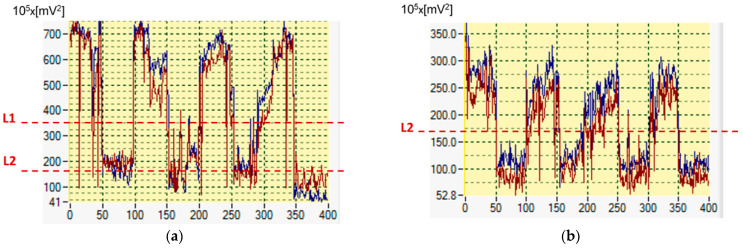
Spectral power P25 + P65 over time from two different human subjects. The subjects were fixating on a target during acquisitions of 0–50, 100–150, 200–250, and 300–350. At all other times, the subjects were fixating on points 1.5° away from the central target. The traces are unfiltered and are from a ***spHWP*** system. Note the different scales. On the power axis (vertical), one division is equivalent to 10^7^ mV^2^.

Another example is presented in Figure 5. The same RBS system as above was employed for data collection, based on Stokes parameter S_1_, and the same data acquisition protocol was used as the one for Figure 4. Yet, this time, data were recorded from two different human test subjects. Again, CF-relevant spectral power P25 + P65 was calculated over time, once for each acquisition epoch of approximately 200 ms duration. The subjects, as before, were fixating on a target during acquisitions of 0–50, 100–150, 200–250, and 300–350. At all other times, the subjects were fixating on points 1.5° away from the central target (off-CF). The spectral traces are unfiltered and are from a ***spHWP*** system.

It can immediately be seen in Figure 5 that although there is good symmetry between the eyes for each subject (unlike what is in Figure 3), the spectral power for subject (a) is more than twice higher than that for subject (b). This presents a problem, because a discrimination threshold at level L1 (about 350 × 10^5^ mV^2^), which works well for subject (a), would not work at all for subject (b). (Please note the different vertical scales in the two plots). Vice versa, discrimination level L2 (about 170 × 10^5^ mV^2^), appropriate for subject (b), would fail completely for subject (a). Clearly, some kind of normalization is needed, which would render the power traces from different subjects, or from one and the same subject under different conditions, comparable—such that a universal threshold for central fixation can be applied.

**Figure 6:**
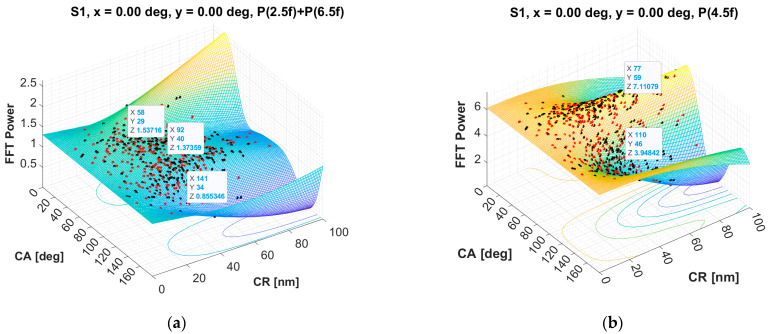
Results from computer modeling of retinal birefringence scanning in the foveal region during central fixation (x = 0°, y = 0°). Presented is Stokes parameter S_1_ as: (**a**) the sum of FFT powers at 2.5*f_s_* and 6.5*f_s_* (both indicative of central fixation), and (**b**) the FFT power at 4.5*f_s_* used in some RBS devices for normalization. The signal powers in both simulations depend on the corneal retardance CR and its azimuth CR. The warping at the edges results from the lack of peripheral data for those extreme values of CR and CA.

A relatively good, but far from perfect, normalization was employed in the ***spHWP*** systems developed earlier in our lab. There, the spectral power P45 (at 4.5*f_s_*) was used for normalization of P25 + P65 [23,24,28,29]. The P45 is widely independent of the direction of gaze, which is why it was employed as a normalizing quantity. But, depending on a number of factors, it may not necessarily change with pupil size, retinal reflectivity, or position of the eye within the exit pupil in the same way as (P25 + P65) does. Also, in many optical designs, P45, as a function of corneal retardance (CR) and corneal azimuth (CA), is not necessarily a flat distribution, as shown in Figure 6 by means of computer modeling. The actual problem is that the cornea, just like the retina, has birefringent properties [30,31,32,33,34,35]. Using a computer model [26,36,37], we were able to simulate the behavior of the signal power at different frequencies, expressed in terms of the scanning frequency *f_s_*. Figure 6 shows the dependence of the signal (Stokes vector S_1_) on corneal birefringence, represented as corneal retardance (CR) and corneal azimuth (CA), both in degrees. The left panel (a) presents the sum of FFT powers at 2.5*f_s_* and 6.5*f_s_* (both indicative of central fixation with an ***spHWP*** system), while the right panel (b) shows the FFT power at 4.5*f_s_*, which is used in some RBS devices for normalization. In all cases, the simulated scanning was strictly around the center of the fovea (mimicking CF). The signal powers in both simulations depend on the corneal retardance CR (*Y*-axis) and its azimuth CA (*X*-axis). It can be seen that while the CF signal (axis Z, left panel, a) is relatively flat for the design at hand, the power at 4.5*f_s_* (axis Z, right panel, b) largely depends on CR and CA. The dots represent real corneal birefringence data from our database [38]—red for the right eye and black for the left eye. The warping (folding) at the edges results from a lack of peripheral data at the extreme values of CR and CA, which impedes perfect interpolation in the right corners of the CR-CA plane.

Figure 6b clearly shows that corneal birefringence affects too much FFT power at 4.5*f_s_* (P45), which makes it impractical for normalization purposes. The distribution revealed in the figure also depends strongly on the specific optical design, in particular, the polarization-sensitive elements in it. In fact, the main goal of the computer modeling carried out in our lab is to keep these distributions flat at the maximum possible signal level (which essentially reduces the S/N ratio) [23,28,37].

In [26], using again a computer RBS model, we have investigated various combinations of frequency powers and ratios that could be included in the CF decision-making, yet none of them has proven to be sufficiently precise, including when off-center frequencies were integrated into a normalization formula. Another concern would be that some 4.5*f_s_* components can penetrate into the channels as back-reflection instrumental noise, inseparable from the P45 coming from the retina, which can adversely impact precision. Last but not least, normalization using the 4.5*f_s_* signal only works with a ***spHWP***, which is more complicated than a ***1f2f*** system (one with no polarization rotator).

## 2. Methods

### 2.1. Hardware Normalization Option

**Figure 7:**
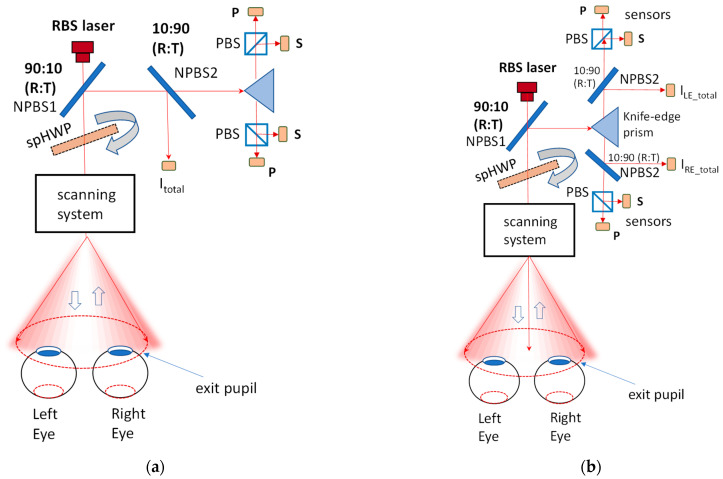
A simplified binocular RBS system. The light reflected from the retina is redirected by a non-polarizing beamsplitter (NPBS1) to a knife-edge prism, which then separates the two eyes. For each eye, a polarizing beamsplitter PBS splits the light into *s*- and *p*-components, which, after measurement by corresponding sensors, are used to build Stokes parameter S_1_. The NPBS2 is used for obtaining a normalization signal based on the total intensity S_0_. The spHWP is a polarization rotator in ***spHWP*** systems. (**a**) Using a common normalization signal for the two eyes. (**b**) Using a normalization for each eye separately to counteract strong asymmetry between the eyes. Not included are light traps, waveplates, mirrors, polarization compensators, a scanning motor, scanning mirrors, etc. Not to scale.

The first normalization method that comes to mind is to use optical and electronic hardware, which would allow measurement of the light returning from the eyes. Figure 7a,b shows a simplified generic RBS system with a normalization signal obtained in addition to (and at the cost of) the main RBS signals. The light reflected from the retina is redirected by a non-polarizing beamsplitter (NPBS1) to a knife-edge prism, which then separates the light coming from the two eyes. For each eye, a polarizing beamsplitter PBS splits the light into *s*- and *p*-components, which, after measurement by corresponding sensors, are used to build Stokes parameter S_1_ = (*s* − *p*). Not included are waveplates, mirrors, lenses, light traps, polarization rotators, polarization compensators, a scanning motor, scanning mirrors, etc. In earlier versions, we built (*s* − *p*) in analog before analog-to-digital conversion. Later, the more accurate technique of building the difference in software after digitization was adopted. This carried the additional advantage of reconstructing S_0_ = (*s + p*) digitally, as will be explained below. The NPBS2 is added with the purpose of obtaining a normalization signal based on a part of the total intensity S_0_. The spHWP is a polarization rotator in ***spHWP*** systems.

In Figure 7a, a part of the optical signal, common for both eyes, is diverted to an additional sensor by means of a second, non-polarizing beamsplitter NPBS2. The normalization signal I_total_ is the same for both eyes. To avoid unnecessary loss of light returned from the eye (potentially resulting in a deteriorated signal-to-noise ratio), a beamsplitter should pass a large portion of the light towards the knife-edge prism (here 90% transmission) and reflect a smaller portion towards the “normalization” sensor (here 10% reflection). The quantity I_total_, equivalent to 10% of Stokes parameter S_0_, is proportional to the total amount of light returning to the sensors and is polarization-independent. It can be used for normalization and accounts for changes in pupil size, retinal reflectivity, and even cataracts. This approach, however, does not consider any imbalance between the eyes, because the S_1_ components from both eyes are normalized by the same denominator I_total_. Should individual normalization for each eye be required, then in the above design, the beamsplitter, along with the additional sensor for I_total_, should be moved between the knife-edge prism and the PBS, one set for each eye. This is suggested in Figure 7b and should work better, yet at the expense of yet another piece of added optics and electronics. Neither of these solutions is optimal. There is also a delicate balance between the amount of light used for obtaining a useful signal S_1_ (already low amplitude and quite noisy) and the diverted amount of light, which is even less and even noisier. Last but not least, adding one or two more ADC channels increases the computational burden, potentially leading to decreased speed in fast RBS systems, especially ones with high data rates, which is a requirement for better time resolution in the detection of clinically important short-lasting episodes of central fixation, such as when testing young children. Expanding the optoelectronics also has another unfavorable effect: increasing the cost and the size of vision screeners, hampering mass production.

For the above reasons, we placed the development of hardware-based normalization on hold and opted for the software normalization described below.

### 2.2. Software Normalization

A pure software normalization solution preferred by us exploits the possibility that the measured *s*- and *p*-polarization components (respectively, Fresnel’s field amplitudes *E*^2^_0x_ and *E*^2^_0y_) can also be used for building an (*s + p*) normalization quantity equivalent to the total power. Although this may sound intuitive, here is a short justification based on [39,40,41]:

(1) $$S_{0}^{2}=S_{1}^{2}+S_{2}^{2}+S_{3}^{2}={\left(E_{0x}^{2}-E_{0y}^{2}\right)}^{2}+{\left(2E_{0x}E_{0y}\cos\delta \right)}^{2}+{\left(2E_{0x}E_{0y}\sin\delta \right)}^{2}$$

(2) $$S_{0}^{2}=E_{0x}^{4}+E_{0y}^{4}-2E_{0x}^{2}E_{0y}^{2}+4E_{0x}^{2}E_{0y}^{2}\left(cos^{2}\delta +sin^{2}\delta \right)$$

(3) $$S_{0}^{2}=E_{0x}^{4}+E_{0y}^{4}+2E_{0x}^{2}E_{0y}^{2}={\left(E_{0x}^{2}+E_{0y}^{2}\right)}^{2}$$

(4) $$S_{0}=E_{0x}^{2}+E_{0y}^{2}=s+p$$

Thus, the digitized *s*- and *p*-signals can first be used to construct S_1_ = (*s* − *p*) and calculate the *P*25 and *P*65 spectral components. Then, the same *s*- and *p*-signals are used for building the combined digital signal (*s + p*), whose spectrum is also calculated to obtain (*P*25 + *P*65)_(s+*p*)_. The latter is then used to normalize the original (*P*25 + *P*65):

(5) $${\left(P25+P65\right)}_{norm}=\frac{{\left(P25+P65\right)}_{\left(s-p\right)}}{{\left(P25+P65\right)}_{\left(s+p\right)}}$$

In real-world applications, it is more practical to include in the denominator also a portion of the off-center frequency powers *P*35 and *P*55, thus improving the formula’s performance for off-center fixation:

(6) $${\left(P25+P65\right)}_{norm}=\frac{{\left(P25+P65\right)}_{\left(s-p\right)}}{{\left(P25+k\left(P35+P55\right)+P65\right)}_{\left(s+p\right)}}$$

A reasonable value for *k* in the above equation would be *k* = 0.5, which takes only half of the para-central powers. With *k* approaching 1.0, the denominator will be approaching S_0_ in the Stokes description.

### 2.3. Test Subjects, Software, and Data Analysis

Eighteen test subjects (ages 26–68) were originally recruited as follows: eight subjects in the age group 20–30 years, five subjects in the age group 31–39 years, one subject was 48 years old, and one was 68 years old. The average age was 32 ± 12.2 years. Data were recorded after obtaining approval from the Johns Hopkins Institutional Review Board (IRB protocol “Continuing Review: CR00049106 for NA_00050844”, Parent Study Name: Retinal Birefringence Scanning of the Eye). Informed consent was obtained from all subjects involved in the study. Of all the volunteers, three were disqualified because of vision issues that were found during an eye exam (myopia; dense vitreous floaters; recent LASIK surgery with significant blur from irregular astigmatism). Data acquisition was accomplished using an RBS of the type shown in Figure 7b above, but without the two beamsplitters NPBS2 and the I_total_ measurement, in order to keep the signal level at a maximum and to avoid unnecessary optic and electronic system extensions. Since the polarization components *s* and *p* were measured separately, the implementation of Equation (6) was straightforward. Each subject used his/her dominant eye. For data collection and analysis, we used our custom software developed in the LabWindows-CVI environment from National Instruments, which offers a versatile C-compiler with rich device drivers and signal processing libraries, enabling fast execution in real time. Statistical analysis (please see Table 1 below) was carried out in MS Excel and MATLAB 2024b.

## 3. Results

**Figure 8:**
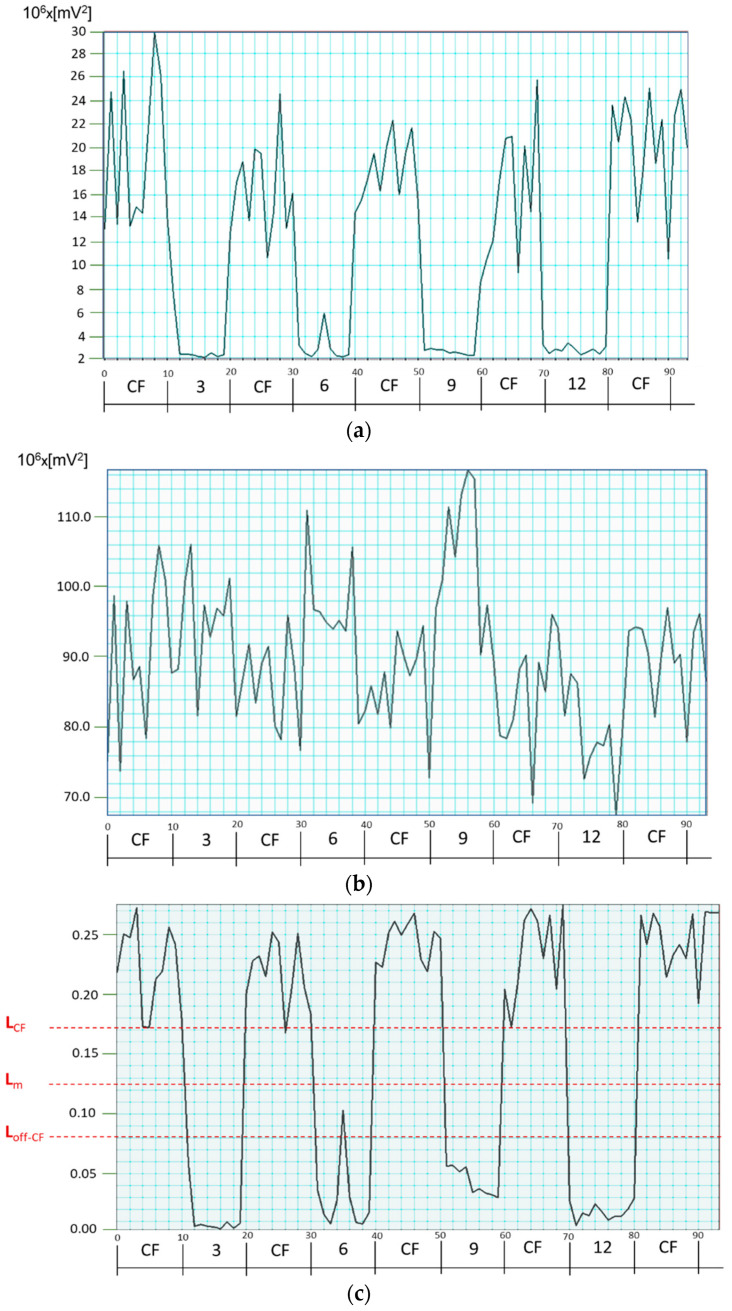
Software normalization of S_1_ using the (s + *p*) quantity (subject #2). (**a**) CF-relevant composite FFT power (P25 + P65)_(s−p)_. (**b**) Total FFT power [P25 + *k*(P35 + P55) + P65]_(s+*p*)_, with *k* = 0.5. (**c**) (P25 + P65)_norm_ according to Equation (6).

Equation (6) is applied to each time sample by finding the joint FFT power, first on the *(s − p)* traces and then on the *(s + p)* traces. Figure 8 shows normalization by (*s + p*) applied to the CF-relevant composite power (P25 + P65) of a ***spHWP*** system on the signals from a typical test subject. Again, the test subject (#2) was asked to fixate on a central target and on four peripheral targets, as in the previous examples. The normalized (P25 + P65)_norm_ was consistently above threshold L_CF_ = 0.170 during central fixation and below L_off-CF_ = 0.080 during fixation on off-center points. A good discrimination threshold for this test subject turned out to be the midlevel L_m_ = 0.125. With this approach, P45 was not used at all, nor were off-center frequency powers included, which we consider a decided advantage.

The results presented in Figure 8 were consistent and similar across all test subjects. This is shown in Table 1. The second column in it shows for each particular subject the threshold above which central fixation can safely be declared. The third column shows the threshold for each subject, below which off-CF should be declared. The fourth column gives the middle between the two levels for each subject, while the fifth column shows a numerically selected common threshold discriminating between CF and off-CF for all test subjects. The next two columns show for each subject the available “reserve” in terms of the distance between the shared common threshold and the individual high or low thresholds, respectively.

A good discrimination threshold turned out to be L_thresh_ = 0.1, which is close to the average midlevel of L_m_ = 0.114 (fourth column in the table). For the discrimination level of L_thresh_ = 0.1, the reserves for high and low are, respectively, 0.071 and 0.044 (standard deviations of 0.055 and 0.021, respectively), as can be seen from the last two columns. Therefore, for the available data, a decision-making threshold of 0.1 separates CF from off-CF without error.

**Figure 9:**
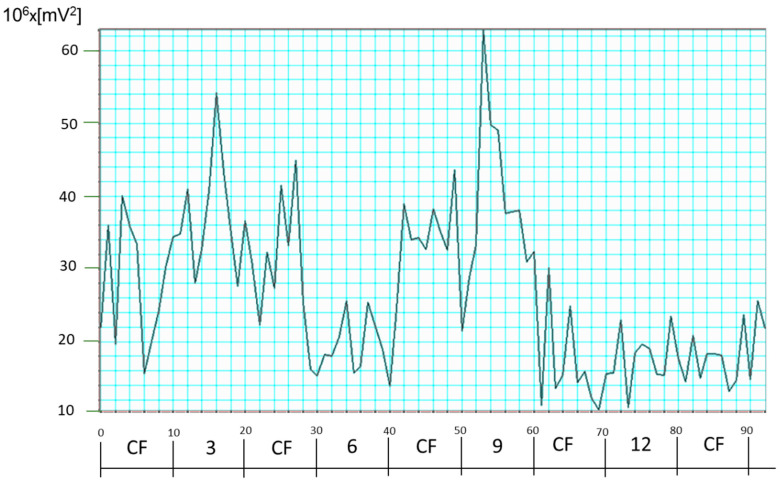
FFT power for 4.5*f_s_* (P45) for test subject #2.

With this approach, P45 was not used at all, nor were off-center frequency powers included in the decision-making, which we consider a decided advantage. In fact, the P45 power for the recording from the same test subject (#2 in the table) varied quite strongly, between 10 × 10^6^ mV^2^ and 60 × 10^6^ mV^2^, as can be seen from the trace for the P45 in Figure 9. This clearly confirms once again that the P45 is not a good candidate for normalization.

## 4. Discussion

Generally, any type of retinal scanning is susceptible to factors like room illumination, pupil size, retinal reflectivity, position of the eye in the device’s exit pupil, patient’s fatigue or inability to ignore distractions, delayed response to instructions by the operator, etc. With pediatric patients these issues are even more expressed. In addition, fixation instability normally occurs from slow drifts and recovery saccades. Consequently, the signals fluctuate, as can be seen in Figure 4, Figure 5, Figure 8 and Figure 9, even when the test subject is attempting to fixate on a well-defined target, with the frequency powers constantly jittering. Time-averaging usually smooths the FFT power traces, but it also considerably deteriorates the time resolution of the system. Proper normalization and careful definition of thresholds are the best way to improve system performance, especially in devices used for screening purposes. Although performed on a limited number of test subjects, this study demonstrates an efficient approach to normalization without adding optical or electronic hardware. Spectral powers at the frequencies of interest in a normalized application are not expected to depend on the optics the way they do with the initially acquired signals. Yet, sample-by-sample normalization, the way it is done here, may not only decrease the amplitude but may also somewhat increase the relative noise. So far, this has not been an issue, judging by the trace in Figure 8c and by the normalized signals obtained from the rest of the test subjects. Should the noise still interfere significantly, the solution will be to apply some local smoothing around each sample, i.e., finite-impulse-response (FIR) low-pass digital filtering with *n* = 2q − 1, q in [2…5], at the cost of a delay of (q − 1) times T, the duration of an FFT epoch (T = 200 ms for the examples in Figure 4 and Figure 5).

A logical question that may arise is, how adequate is the data acquired from the test subject and would it be applicable to a pediatric population? Indeed, some studies show that there is a slight yet significant decrease in the *thickness* of the retinal nerve fiber layer (RNFL) and the Henle fiber layer (HFL) with age. For example, in [42], the authors report a drop in RNFL thickness from 100 μm for age < 20 years (mean 11.2 years, standard deviation 3.9 years) to 92 μm for age > 50 years. Age-related RNFL loss was not uniform in all the quadrants, with maximum loss in the superior quadrant, and it seemed to reach a maximum after the age of 50 years. For the HFL, a lower thickness decline was reported: −0.07 μm/year, i.e., ~3.5 μm over 50 years [43]. It is more interesting to see how age impacts *phase retardation* in polarization-sensitive systems. Using the GDx, the authors in [44] studied 10 males and 10 females from each decade from the third decade to the eighth. The authors correctly state that the “radially symmetric pattern of the axons leads to systematic variation of phase retardation along a ring concentric with the fovea with modulation that is described by a sine function, but with twice the frequency of the ring”. They modeled the amplitude of the 2f phase retardation with the following equation:

*y* = *a*(*sin*(*x* + *b*)) + *c* (7)

where coefficient *a* represents the amplitude of the intensity, *b* is the phase offset, and *c* is a generalized offset term. Upon analyzing the entire sample of 120 eyes, the authors found a drop of *a* by 0.086/year for eccentricity (distance from the foveal center) of 1.25°. In accordance with this, and as can be seen in their Figure 2, phase retardation is expected to drop by ca. 23% from 25 to 65 years of age, or by 7.3% from 25 to 36 years of age, which is where 87% of our data is (13 out of 15 subjects). In the other direction, going backward from the age of 25, if the same trend were to hold true, one could expect an increase of phase retardation by 13% between 25- and 5-year-olds. Even so, for a system that is based on normalization, such as ours, this does not present a serious problem.

A more recent study, this time by the group at the Medical University of Vienna, using polarization-sensitive optical coherence tomography (PS-OCT) on 150 healthy subjects (20–39 years), did **not** find a reduction of HFL retardation with age [45]. The authors used an enhanced PS-OCT system that also incorporated an updated retinal tracker to counter involuntary eye movements. Their system achieved an axial optical resolution of 4.2 μm, a lateral resolution of ~20 μm, and a sensitivity of 98 dB. In addition, each subject was imaged three times on two different study days. Various metrics of the retardation pattern, such as maximum retardation, the eccentricity of the maximum retardation, the retardation area under the curve (AUC) between 1° and 8° eccentricity, and the mean retardation per annulus, were statistically analyzed. The difference between younger and older subjects was **not** significant for any of these measures.

The above is in agreement with the fact that our previous studies have shown no significant differences between RBS signals collected from populations of toddlers to middle-aged subjects.

## 5. Conclusions

In summary, we propose a novel self-consistent method for normalization of RBS signals and demonstrate its efficiency with human data. The method helps overcome dependence on changes in pupil size, variations in retinal reflectivity, position within the exit pupil of the device, and even signal degradation from cataracts. It is straightforward to implement, does not need any additional hardware, and does not need high-precision or repetitive adjustment. The method holds promise to significantly improve the precision of RBS systems, especially those used for examining eye alignment in pediatric vision screeners.

## Figures and Tables

**Table 1:** Numerically normalized data for all test subjects. A threshold of 0.1 can separate CF from off-CF without error and with a sufficient reserve on both sides.

				Selected Threshold		
Subject Number	Lowest Signal Power for CF (High)	Highest Signal Power for Off-CF (Low)	Middle Line	0.1	Upper Reserve	Lower Reserve
1	0.115	0.050	0.083		0.015	0.050
2	0.170	0.080	0.125		0.070	0.020
3	0.105	0.025	0.065		0.005	0.075
4	0.118	0.020	0.069		0.018	0.080
5	0.127	0.070	0.099		0.027	0.030
6	0.180	0.023	0.102		0.080	0.077
7	0.110	0.040	0.075		0.010	0.060
8	0.240	0.050	0.145		0.140	0.050
9	0.150	0.080	0.115		0.050	0.020
10	0.250	0.075	0.163		0.150	0.025
11	0.220	0.060	0.140		0.120	0.040
12	0.180	0.060	0.120		0.080	0.040
13	0.240	0.080	0.160		0.140	0.020
14	0.240	0.070	0.155		0.140	0.030
15	0.125	0.060	0.093		0.025	0.040
	0.171	0.056	0.114	<=Averages=>	0.071	0.044
	0.055	0.021	0.034	<=StDevs=>	0.055	0.021

## Data Availability

Data underlying the results presented in this paper are not publicly available at this time but may be obtained from the authors upon reasonable request.
